# Neuropsychological differences between treatment-resistant and treatment-responsive schizophrenia: a meta-analysis

**DOI:** 10.1017/S0033291721004128

**Published:** 2022-01

**Authors:** Edward Millgate, Olga Hide, Stephen M Lawrie, Robin M Murray, James H MacCabe, Eugenia Kravariti

**Affiliations:** 1 Department of Psychosis Studies, Institute of Psychiatry, Psychology and Neuroscience, King's College London, London, UK; 2 Division of Psychiatry, University of Edinburgh, Edinburgh, UK

**Keywords:** treatment-resistant schizophrenia, neuropsychological deficits, verbal memory and learning, language functions, meta-analysis

## Abstract

Antipsychotic treatment resistance affects up to a third of individuals with schizophrenia. Of those affected, 70–84% are reported to be treatment resistant from the outset. This raises the possibility that the neurobiological mechanisms of treatment resistance emerge before the onset of psychosis and have a neurodevelopmental origin. Neuropsychological investigations can offer important insights into the nature, origin and pathophysiology of treatment-resistant schizophrenia (TRS), but methodological limitations in a still emergent field of research have obscured the neuropsychological discriminability of TRS. We report on the first systematic review and meta-analysis to investigate neuropsychological differences between TRS patients and treatment-responsive controls across 17 published studies (1864 participants). Five meta-analyses were performed in relation to (1) executive function, (2) general cognitive function, (3) attention, working memory and processing speed, (4) verbal memory and learning, and (5) visual−spatial memory and learning. Small-to-moderate effect sizes emerged for all domains. Similarly to previous comparisons between unselected, drug-naïve and first-episode schizophrenia samples *v.* healthy controls in the literature, the largest effect size was observed in verbal memory and learning [*dl* = −0.53; 95% confidence interval (CI) −0.29 to −0.76; *z* = 4.42; *p* < 0.001]. A sub-analysis of language-related functions, extracted from across the primary domains, yielded a comparable effect size (*dl* = −0.53, 95% CI −0.82 to −0.23; *z* = 3.45; *p* < 0.001). Manipulating our sampling strategy to include or exclude samples selected for clozapine response did not affect the pattern of findings. Our findings are discussed in relation to possible aetiological contributions to TRS.

## Introduction

Up to a third of individuals with schizophrenia show resistance to antipsychotic treatment (Elkis & Buckley, 2016; Lally, Gaughran, Timms, & Curran, 2016b; Mørup, Kymes, & Oudin Åström, 2020; Stokes et al., 2020), i.e. they do not respond adequately to two or more trials of antipsychotic medication, each lasting 4–6 weeks, at doses in at least the mid-point of the licensed therapeutic range (NICE guidelines; National Institute for Health and Care Excellence, 2014). In comparison to treatment responsive patients, those with treatment-resistant schizophrenia (TRS) tend to experience multiple symptomatic relapses, are exposed to higher doses of antipsychotic medication, and show poor functional recovery (Chan et al., 2021; Iasevoli et al., 2016).

Evidence of glutamatergic rather than dopaminergic abnormalities in TRS **(**Demjaha et al., 2014; Gillespie, Samanaite, Mill, Egerton, & MacCabe, 2017; Goldstein, Anderson, Pillai, Kydd, & Russell, 2015; Mouchlianitis et al., 2016) raises the possibility that the disorder is categorically distinct from treatment-responsive schizophrenia (Gillespie et al., 2017). Running counter to this possibility, clozapine surpasses other antipsychotics in improving total and positive symptoms in both TRS and treatment-responsive patients (Mizuno, McCutcheon, Brugger, & Howes, 2020), supporting arguments against an illness subtype that responds specifically to clozapine (Mizuno et al., 2020). A possibility that merits exploration is that TRS is aetiologically continuous with treatment-responsive schizophrenia but occupies a more extreme position in a continuum of neurodevelopmental impairment. In support of this hypothesis, 70–84% of patients with treatment-resistant psychosis are reported to be resistant from the first episode (Demjaha et al., 2017; Lally et al., 2016a). In addition, some of the strongest predictors of poor therapeutic response in schizophrenia are the same as the defining features of what has been termed ‘neurodevelopmental’ schizophrenia: male sex, younger age at disease onset, poor premorbid adjustment, and longer duration of untreated illness (Carbon & Correll, 2014; Murray, O'Callaghan, Castle, & Lewis, 1992).

Understanding the aetiological and neurobiological mechanisms of TRS is important for developing personalised medicine, for ensuring early detection, and for initiating timely and appropriate treatment. The gold standard treatment for TRS is clozapine (Kane, Honigfeld, Singer, & Meltzer, 1988), with early pharmacological intervention improving functional outcomes in ~80% of those treated (John, Ko, & Dominic, 2018; Üçok et al., 2015; Yoshimura, Yada, So, Takaki, & Yamada, 2017). In contrast, a 3-year delay in commencing clozapine reduces response rates to only ~30% (Yoshimura et al., 2017). Notwithstanding this evidence and treatment guidelines, antipsychotic polypharmacy and high doses are commonly used prior to clozapine, which is initiated with a mean delay of 4 years (Howes et al., 2012).

Neuropsychological investigations can offer important insights into the nature, origin and pathophysiology of TRS. To date, a number of studies have reported deficits in verbal intelligence and memory, attention, working memory, visuospatial processing, and sensorimotor function in TRS patients compared to treatment-responsive controls (Anderson, McIlwain, Kydd, & Russell, 2015; Bourque *et al*. 2013; de Bartolomeis *et al*. 2013; Frydecka, Beszłej, Gościmski, Kiejna, & Misiak, 2016; Huang *et al*. 2020; Joober et al., 2002; Lin, Chan, Peng, & Chen, 2019). However, inconsistent findings, as well as methodological variability and limitations in a largely emergent field of research make it difficult to elaborate on the neuropsychological profile of TRS and to discern its discriminability compared to schizophrenia at large. The only neuropsychological investigation to date to directly compare longitudinally characterised treatment-resistant and treatment-responsive patients at their first episode of psychosis found relative deficits in language functions in the former group (Kravariti et al., 2018). As such functions are largely reflective of premorbid ability, the authors concluded that treatment-resistant psychosis is likely to represent a severe variant of psychosis, embedded in aberrant neurodevelopmental processes (Kravariti et al., 2018).

We report on the first systematic review and meta-analysis to investigate and quantify differences in neuropsychological performance between patients with TRS and those responsive to antipsychotic treatment. Based on previous findings, we predicted that TRS patients would perform worse than treatment-responsive patients, and that verbal functions would yield the largest effect sizes.

## Methods

### Search strategy

Records were accessed from PsycINFO (1806 to October Week 3 2020), Ovid MEDLINE(R) (1946 to October 22nd 2020) and Web of Science on 24th October 2020. Search terms were selected using a PICO framework. Search terms which were exploded in MEDLINE and PsychINFO are indicated with ‘^a^’ in the description below, with asterisks (*) indicating a wildcard search term. This meta-analysis was registered on PROSPERO (CRD42019147035).

**Fig. 1.**
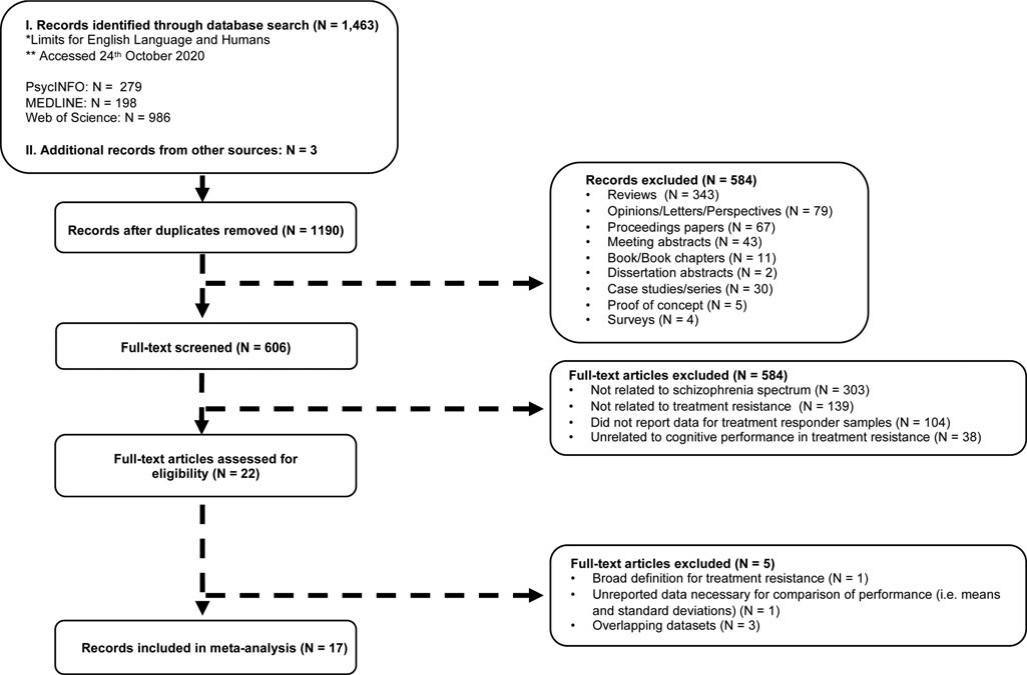
A PRISMA based flow diagram of the literature search strategy and study selection.

The following search terms were used: (Treatment-resistant schizophrenia *OR* TRS *OR* Treatment-refractory schizophrenia *OR* Antipsychotic-resistant OR Antipsychotic-refractory) *AND* (Cognit* *OR* Neuropsy* *OR* Executive function (^a^) *OR* Memory (^a^) *OR* Intelligence (^a^) *OR* Attention (^a^) *OR* Awareness (^a^) *OR* Learning (^a^). Additional publications (*N* = 3) were sourced from Google Scholar, PsycINFO, and Web of Science through searching ‘*Treatment-resistance.*’ Figure 1 provides a summary of the literature search strategy, using the PRISMA Group's guidelines for systematic reviews and meta-analyses (Liberati et al., 2009; Moher, Liberati, Tetzlaff, Altman, & Group, 2009). The PRISMA checklist is presented in online Supplementary Table S1.

### Inclusion/exclusion criteria

Studies were included in the systematic review and meta-analysis, if they:

1. Reported neuropsychological findings from individuals with treatment-resistant, and controls with treatment-responsive, schizophrenia-spectrum disorders.
2. Defined schizophrenia-spectrum disorders according to the diagnostic criteria of the International Classification of Diseases (ICD) or the Diagnostic and Statistical Manual of Mental Disorders (DSM) classification systems (online Supplementary Table S2).
3. Adopted one of two definitions of treatment resistance: i. not responding adequately to antipsychotic treatment, despite the use of two or more different antipsychotics, each lasting 4–6 weeks, at doses in at least the mid-point of the licensed therapeutic range (NICE guidelines; National Institute for Health and Care Excellence, 2014), or ii. Currently treated with clozapine (online Supplementary Table S2).

With the exception of two studies (Anderson et al., 2015; Lin et al., 2019), all publications which met inclusion criteria comprised participants unselected for clozapine response. Anderson et al. (2015) and Lin et al. (2019) included discrete subgroups of clozapine-responsive and clozapine-resistant (termed ultra-resistant) subgroups. To address the theoretical risk of inflated (ultra-resistant) or deflated (clozapine-responsive) effect sizes of cognitive deficits compared to unselected TRS samples, we adopted the conservative strategy of excluding the two ultra-treatment resistant subgroups from our main analyses. This approach maximised the analytic sample (the clozapine-responsive subgroups from both studies were retained in the main analysis), whilst cautiously biasing effect sizes towards conservative rather than inflated estimates (by excluding ultra-resistant samples). To examine if manipulating our sampling strategy would have any impact on our findings, the analysis was performed before (main analysis) and after (sensitivity analysis) (a) excluding clozapine-responsive samples (Anderson et al., 2015; Lin et al., 2019); (b) adding clozapine-resistant samples (Anderson et al., 2015; Lin et al., 2019).

As shown in Fig. 1, reviews, opinions, proceeding papers, meeting abstracts, letters, proof of concept studies, and case studies were not included in the study. Of published studies with overlapping participant samples, the largest study, or the one reporting on the largest set of neuropsychological findings, was included. This approach resulted in the exclusion of three publications (de Bartolomeis et al., 2018; Iasevoli et al., 2017, 2018b). In cases of partial reporting on essential findings (e.g. means and standard deviations of composite scores rather than individual variables), two attempts were made to obtain data from corresponding authors before excluding the paper. Data were received for four studies (Kravariti et al., 2018; Lawrie et al., 1995; Legge et al., 2019; Vanes, Mouchlianitis, Collier, Averbeck, & Shergill, 2018a).

### Data extraction

For our main analysis (see below), 41 cognitive tasks were grouped into five primary cognitive domains by E.M. & E.K. based on each task's underpinning theoretical construct and earlier groupings in the literature (Fatouros-Bergman, Cervenka, Flyckt, Edman, & Farde, 2014; Fett, Viechtbauer, Penn, van Os, & Krabbendam, 2011; Fioravanti, Carlone, Vitale, Cinti, & Clare, 2005): (1) executive function, (2) general cognitive function, (3) attention, working memory and processing speed, (4) verbal memory and learning, and (5) visual−spatial memory and learning (Table 1). Three of the 41 tasks (National Adult Reading Test, Phonological Verbal Fluency, Semantic Verbal Fluency) were also included in our sub-analysis of language-related functions (see below); the latter further included Vocabulary (which did not feature in the main analysis) (Table 1). The following information was extracted from each publication by two independent investigators (E.M. & O.H.): author names, publication year, diagnostic criteria for schizophrenia-spectrum disorders, the definition of TRS, and, for each of the TRS and treatment-responsive groups, number of cases, mean age, number of males, mean age of illness onset, mean duration of illness, mean chlorpromazine equivalents, mean years of education, mean positive and negative symptom scale scores, as well as means and standard deviations of cognitive tasks (online Supplementary Table S4).

**Table 1.** Cognitive tasks contributing to the main analysis of five primary cognitive domains and the sub-analysis of language-related functions from across primary cognitive domains

Cognitive domain	Cognitive task (subtest)	Publication
Main analysis^1^		
Executive function	BACS Category Instances Task	de Bartolomeis et al. (2013); Iasevoli et al. (2018a)
	BACS Tower of London	de Bartolomeiset al. (2013); Iasevoli et al. (2018a)
	BRCCB Information Processing Speed	Anderson et al. (2015)
	BRCCB Information Processing Efficiency	Anderson et al. (2015)
	BRCCB Verbal Fluency	Anderson et al. (2015)
	Letter-Number Span	Kravariti et al. (2018); Rakitzi and Georgila (2019)
	Neuropsychological Assessment Battery: Mazes	Huang et al. (2020)
	Modified Wisconsin Card Sorting Test	Smith, Kadewari, Rosenberger, and Bhattacharyya (1999)
	Stroop Test (incongruent trial)	Lawrie et al. (1995); Frydecka et al. (2016); Vanes et al. (2018a)
	Trail Making B	Smith et al. (1999); Frydecka et al. (2016); Kravariti et al. (2018)
	Phonological Verbal Fluency	Lawrie et al. (1995); Smith et al. (1999); Frydecka et al. (2016); Kravariti et al. (2018)
	Semantic Verbal Fluency	Smith et al., 1999; Frydecka *et al*., 2016; Kravariti *et al*., 2018
General cognitive function	MCCB Composite	Huang *et al*. (2020)
	Mini Mental State Examination	Lawrieet al. (1995); Joober et al. (2002); Gong et al. ( 2020)
	National Adult Reading Test	Lawrie et al. (1995); Kravariti et al. (2018); Legge et al. (2019)
	Quick Test IQ	Lawrie et al. (1995)
	Wechsler Abbreviated Scale of Intelligence (abbreviated; two-subsets)	Vanes, Mouchlianitis, Wood, and Shergill (2018b)
	WAIS-R Full Scale IQ	White et al. (2016); Kravariti et al. (2018); Rakitzi and Georgila (2019)
Attention, working memory and processing speed	BACS Digit Sequencing Task	de Bartolomeis et al. (2013); Iasevoli et al. (2018a)
	BACS Symbol Coding Task	de Bartolomeis et al. (2013); Iasevoli et al. (2018a)
	BRCCB Working Memory	Anderson et al. (2015)
	BRCCB Sustained Attention	Anderson et al. (2015)
	Continuous Performance Test	Iasevoli et al. (2018a); Rakitzi and Georgila (2019); Lin et al. (2019)
	MCCB Attention/Vigilance	Huang et al. (2020)
	MCCB Working Memory	Huang et al. (2020)
	Stroop Test (congruent trial)	Frydecka et al. (2016)
	Trail Making A	Smith et al. (1999); Frydecka et al. (2016); Kravariti et al. (2018)
	WAIS-R Digit Symbol Coding Test	Lawrie et al. (1995); Frydecka et al. (2016); Kravariti et al. (2018)
	WAIS-R Digit Span Backward	Lawrie et al. (1995); Smith et al. (1999); Frydecka et al. (2016)
	WAIS-R Digit Span Forward	Lawrie et al. (1995); Smith et al. (1999); Frydecka et al. (2016)
Verbal memory and learning	BACS List Learning Task	de Bartolomeis et al. (2013); Iasevoli et al. (2018a)
	BRCCB Verbal Learning and Memory	Anderson et al. (2015)
	Greek Verbal Memory Test	Rakitzi and Georgila (2019)
	MCCB Hopkins Verbal Learning Test – Revised	Huang et al. (2020)
	Rey Auditory Verbal Learning Test	Frydecka et al. (2016); Kravariti et al. (2018)
	Rivermead Behavioural Memory Test	Lawrie et al. (1995)
Visual−spatial memory and learning	Brief Visuospatial Memory Test-Revised	Iasevoli et al. (2018a)
	BRCCB Visuospatial Learning and Memory	Anderson et al. (2015)
	CANTAB Spatial Recognition	Lawrie et al. (1995)
	MCCB Visual Learning	Huang et al. (2020)
	WMS-R Visual Reproduction Trials	Kravariti et al. (2018)
Sub-analysis of language-related functions^2^		
Language function	National Adult Reading Test^a^	Lawrie *et al*. (1995); Kravariti et al. (2018); Legge et al. (2019)
	Phonological Verbal Fluency^b^	Lawrie *et al*. (1995); Smith et al. (1999); Frydecka et al. (2016); Kravariti et al. (2018)
	Semantic Verbal Fluency^b^	Smith *et al*. (1999); Frydecka et al. (2016); Kravariti et al. (2018)
	WAIS-III Vocabulary^c^	Bourque et al. (2013); Kravariti et al. (2018)

*Abbreviations: BACS*, Brief Assessment of Cognition in Schizophrenia; *BRCCB*, Brain Resource Centre Cognitive Battery; *CANTAB*, Cambridge Neuropsychological Testing Automated Battery; *MCCB*, MATRICS Consensus Cognitive Battery; *WAIS-III*, Wechsler Adult Intelligence Scale-Third edition; *WAIS-R*, Wechsler Adult Intelligence Scale-Revised; *WMS-R*, Wechsler Memory Scale-Revised.

^a^Extracted from the ‘General cognitive domain’ of the main analysis; ^b^extracted from the ‘Executive function’ domain of the main analysis; ^c^was not included in the main analysis.

1 The main analysis included all treatment-resistant schizophrenia (TRS) samples across publications, except for the clozapine-resistant samples in Anderson et al., 2015 and Lin et al., 2019. Both clozapine-resistant samples were added to the main analytic sample as part of our sensitivity analysis (see online Supplementary Fig. S3).

2 The sub-analysis focused selectively on language-related functions that were extracted from across the primary cognitive domains of the main analysis, in addition to Wechsler Vocabulary; the latter task was only included in the sub-analysis and did not feature in the main analysis.

Quality assessments for each publication were made using the *Quality Assessment Tool for Observational Cohort and Cross-Sectional Studies* and *Quality Assessment of Controlled Intervention Studies* tools from the National Heart, Lung and Blood Institute (NIH, 2014a). Each publication was independently rated by E.M and O.H, providing a ‘*yes*’, ‘*no*,’ ‘*not applicable*,’ ‘*cannot determine*,’ or ‘*not reported*’ response to each of fourteen statements. An overall quality rating (*good, fair or poor*) was derived based on these responses (online Supplementary Table S3).

### Main analysis: Meta-analysis of five primary cognitive domains

Data were analysed in relation to five cognitive domains using the *metan* (Harris et al., 2008), *metaan* (Kontopantelis & Reeves, 2010), *metabias* (Harbord, Harris, Sterne, & Steichen, 2009) and *metafunnel* (Sterne, 2003) commands in STATA/SE Version 15. The *metaan* command runs meta-analyses off the saved estimates from *metan* using a restricted maximum likelihood model (REML) and providing *I*^2^ and Cochrane *Q* estimates for heterogeneity. The REML method reduces the likelihood of both positive and negative biases and has been recommended over eight other methods in a recent comparison of nine different heterogeneity variance estimators using simulated meta-analysis data (Langan et al., 2019). *Z* statistics, *p* values and 95% confidence intervals (CIs) were estimated for the effect sizes following Altman & Bland's (2011) recommendations. Where two or more tasks from the same study contributed to the same cognitive domain, estimates from the *metan* command were used to create a within-study weighted average prior to *metaan*. This step was undertaken to preserve the independence of participant samples within each cognitive domain. Findings of significant heterogeneity in any cognitive domain were followed by meta-regressions using the *metareg* command (Harbord & Higgins, 2008) to examine potential demographic (age, sex, and years of education), clinical (duration of illness, age at illness onset, positive and negative symptom ratings) and medication (chlorpromazine equivalents) sources of heterogeneity. Differences between groups in these variables were included as individual predictors in the meta-regression models.

### Sub-analysis: Meta-analysis of language-related functions from across primary cognitive domains

Earlier findings from our research group (Kravariti et al., 2018) suggested that patients with a first episode of psychosis, who were later found to be treatment resistant, were impaired in verbal intelligence and fluency, but in no other composite scores, relative to their treatment-responsive counterparts. To examine the salience of language-related functions in the neuropsychological profile of TRS, in a second step, we selectively extracted tasks with a prominent language processing component from across the five primary cognitive domains (General cognitive function: National Adult Reading Test; Executive function: Phonological Verbal Fluency; Semantic Verbal Fluency), further adding Wechsler Vocabulary (which did not feature in the main analysis) to conduct a separate, language-focused meta-analysis (Table 1).

### Sensitivity analysis: Clozapine response

Forty to 70% of TRS patients respond partially or poorly even to clozapine (Farooq, Choudry, Cohen, Naeem, & Ayub, 2019; Potkin et al., 2020; Siskind, Siskind, & Kisely, 2017) and are termed ultra-resistant. For the latter group, non-pharmacological augmentation strategies, such as electroconvulsive therapy (ECT) and transcranial direct-current stimulation (tDCS) are shown to hold considerable promise (Lindenmayer et al., 2019; Moulier, Krir, Dalmont, Guillin, & Rothärmel, 2021). These critical differences in treatment response have been proposed to correspond to three sub-types of schizophrenia: antipsychotic-responsive, clozapine-responsive and clozapine-resistant (Farooq et al., 2019).

A direct comparison of neuropsychological performance across the three subtypes would critically enhance the resolution and impact of our analysis. However, all but two studies (Anderson et al., 2015; Lin et al., 2019) included participants unselected for clozapine response, preventing such comparison. We instead examined the sensitivity of our analysis to (a) excluding the clozapine-responsive subgroups of both studies (these were included in the main analysis for the reasons outlined in ‘Inclusion/exclusion criteria’); (b) adding the ultra-treatment resistant subgroups from both studies. To approximate the predominant (undifferentiated) sampling strategy in the literature (and to preserve sample independence), the latter addition was performed by averaging the cognitive scores across the clozapine-responsive and the clozapine-resistant subgroups from each study (rather than including two discrete TRS samples from each study).

## Results

### Study characteristics

A total of 17 studies (16 observational, one experimental) made up our analytic sample (Fig. 1, Table 2). Table 1 lists the cognitive tasks employed across publications in relation to each of the five primary cognitive domains (main analysis) and in relation to the language-related functions (sub-analysis). The descriptive characteristics of the study samples are presented in Table 2. The 17 publications contributed 1864 participants (939 TRS) and 77 discrete comparisons in cognitive performance between TRS and treatment-responsive participants to the main analysis (online Supplementary Table S4), and 1129 participants (584 TRS) and 10 discrete comparisons in cognitive performance between TRS and treatment-responsive participants to the language sub-analysis (online Supplementary Table S4). The sensitivity analyses included 1678–1933 participants (857–994 TRS) (online Supplementary Figs S2 and S3). Only baseline / pre-intervention data were included from the experimental study by Rakitzi and Georgila, 2019.

**Table 2.** Descriptive characteristics of treatment-resistant and treatment-responsive schizophrenia samples in the 17 publications

		Treatment responsive								Treatment resistant (TRS)							
Publication	Total sample size	*n* (male)	Age	Age of onset	DOI	CPZEs	Years of education	Positive symptom score (*z*)	Negative symptom score (*z*)	*n* (male)	Age	Age of onset	DOI	CPZEs	Years of education	Positive symptom score (*z*)	Negative symptom score (*z*)
Anderson et al. (2015)	36	16 (13)	32.60	–	10.30	390.00	12.5	0.15	0.31	20 (15)	33.2	–	12.9	435	11.1	−0.60	0.02
Bourque et al. (2013)	43	23 (10)	30.21	23.19	6.69	332.54	11.87	0.95	0.80	20 (12)	33.80	21.68	12.21	891.67	11.40	−0.13	−0.03
de Bartolomeis et al. (2013)	41	22 (19)	35.95	21.75	13.65	413.90	13.86	–	–	19 (17)	36.31	20.68	17.32	679.30	12.47	–	–
Frydecka et al. (2016)	85	32 (11)	35.44	24.43	10.84	319.70	13.8	0.97	0.85	53 (24)	37.17	24.19	13.00	590.39	13.1	0.42	0.46
Gong et al. ( 2020)	53	20 (11)	27.50	–	1.70	–	–	2.07	1.21	33 (24)	31.50	–	8.3	–	–	1.17	0.35
Huang et al. (2020)	86	43 (28)	46.50	25.10	22.10	470.60	12.30	−0.24	0.08	43 (28)	48.50	25.41	25.30	745.90	0.58	0.30	0.30
Iasevoli et al. (2018a)	60	32 (16)	35.09	22.78	12.09	369.45	12.91	0.71	0.88	28 (19)	39.79	21.79	18.00	539.93	12.21	0.15	0.53
Joober et al. (2002)	75	36 (23)	39.00	24.00	15.50	669.00	11.6	−1.57	−1.36	39 (34)	36.00	17.90	18.40	1340.00	11.4	−1.79	−1.23
Kravariti et al. (2018)	139	109 (60)	30.14	30.23	–	334.66	12.6	–	−1.33	30 (23)	26.00	25.41	–	427.81	12.10	–	−1.20
Lawrie et al. (1995)	40	20 (10)	36.50	–	11.61	268.00	14.0	−1.56	−1.40	20 (10)	34.70	–	12.57	902.00	10.9	−1.74	−1.30
Legge et al. (2019)	817	361 (231)	44.71	27.03	–	–	13.22	–	–	456 (306)	42.09	23.10	–	–	12.75	–	–
Lin et al. (2019)	150	102 (57)	43.50	25.70	17.30	385.50	10.4	–	–	48 (27)	45.60	22.50	23.00	458.30	9.90	–	–
Rakitzi and Georgila (2019)	72	39 (28)	32.77	–	5.55	443.65	–	1.56	1.75	33 (20)	32.34	–	5.80	624.79	–	1.09	1.05
Smith et al. (1999)	45	20 (12)	39.40	22.50	15.40	1300.20	12.70	−1.17	0.69	25 (13)	43.80	19.20	25.10	1005.60	11.30	1.79	2.25
Vanes et al. (2018a)	42	21 (18)	41.30	27.70	14.10	280.30	–	−0.27	−0.10	21 (18)	41.50	26.00	15.50	383.50	–	0.27	0.02
Vanes et al. (2018b)	42	21 (18)	41.30	27.70	14.10	280.30	–	−0.27	−1.00	21 (18)	41.50	26.00	15.50	383.50	–	0.27	0.02
White et al. (2016)	38	22 (19)	37.55	25.57	11.86	281.68	–	0.31	0.45	16 (12)	36.69	21.34	15.47	764.06	–	−0.01	0.02

*Abbreviations: CPZEs*, chlorpromazine equivalents*; DOI*, duration of illness; *TR*, treatment responder; *TRS*, treatment-resistant schizophrenia.

*Note*: All values reported are mean values unless indicated otherwise.

The specific list and number of publications that contributed analytic data (i.e. discrete comparisons in cognitive performance between treatment-responsive and TRS participants) to the main analysis and sub-analysis is listed in online Supplementary Table S4, and included 10 publications (24 comparisons) for executive function, nine publications (12 comparisons) for general cognitive function, 10 publications (24 comparisons) for attention, working memory and processing speed, eight publications (12 comparisons) for verbal memory and learning and five publications (five comparisons) for visual−spatial memory and learning (online Supplementary Table S4). Six publications contributed analytic data (10 comparisons) to the sub-analysis of language-related functions (online Supplementary Table S4).

### Main analysis: Meta-analysis of five primary cognitive domains

Table 3 illustrates the REML findings and heterogeneity estimates for the main analysis (five primary cognitive domains) and the sub-analysis (language-related functions). TRS patients scored lower than treatment-responsive patients, with effect sizes ranging from small to moderate (based on Cohen's thresholds: 0.2 = small, 0.5 = medium, 0.8 = large; Cohen, 1988). Effect sizes were statistically significantly different from 0, except for executive function and visual−spatial memory and learning (Table 3). The largest effect size (moderate) emerged for verbal memory and learning (*dl* = −0.53). The remaining cognitive domains gave rise to small effects (*dl* = −0.27 to −0.38) (Table 3).

**Table 3.** Meta-analyses of performance differences between treatment-resistant and treatment-responsive schizophrenia samples across publications

Cognitive domain	No. of studies	Treatment responsive (*n*)	TRS (*n*)	Overall effect (REML)	L95% CI	U95% CI	*z*	*p* value	Cochrane *Q*	*I*^2^ (%)	*T*^2^
Main analysis^1^											
Executive function	10	353	290	−0.32	−0.58	−0.07	2.46	0.068	20.51, *p* = 0.015	56.26	0.09
General cognitive function	9	671	691	−0.29	−0.43	−0.14	3.92	0.001	13.01, *p* = 0.112	17.59	0.01
Attention, working memory and processing speed	10	432	373	−0.38	−0.52	−0.23	5.14	< 0.001	7.82, *p* = 0.552	–	–
Verbal memory and learning	8	306	243	−0.53	−0.76	−0.29	4.42	< 0.001	13.25, *p* = 0.066	40.10	0.05
Visual−spatial memory and learning	5	212	154	−0.27	−0.50	−0.05	2.35	0.085	2.69, *p* = 0.612	–	–
Sub-analysis of language-related functions^2^											
Language-related functions	6	564	603	−0.53	−0.82	−0.23	3.45	< 0.001	13.96, *p* = 0.016	64.17	0.08

*Abbreviations and notes: I*^2 (%)^, proportion of observed variance of effect sizes; *LCI*, lower confidence interval; *REML*, Restricted maximum likelihood model; *T*^2^, measure of dispersion from true effect size; *UCI*, upper confidence interval.

1 The main analysis included all treatment-resistant schizophrenia (TRS) samples across publications, except for the clozapine-resistant samples in Anderson et al., 2015 and Lin et al., 2019. Both clozapine-resistant samples were added to the main analytic sample as part of our sensitivity analysis (see online Supplementary Fig. S3).

2 The sub-analysis focused selectively on language-related functions that were extracted from across the primary cognitive domains of the main analysis, in addition to Wechsler Vocabulary; the latter task was only included in the sub-analysis and did not feature in the main analysis.

**Fig. 2.**
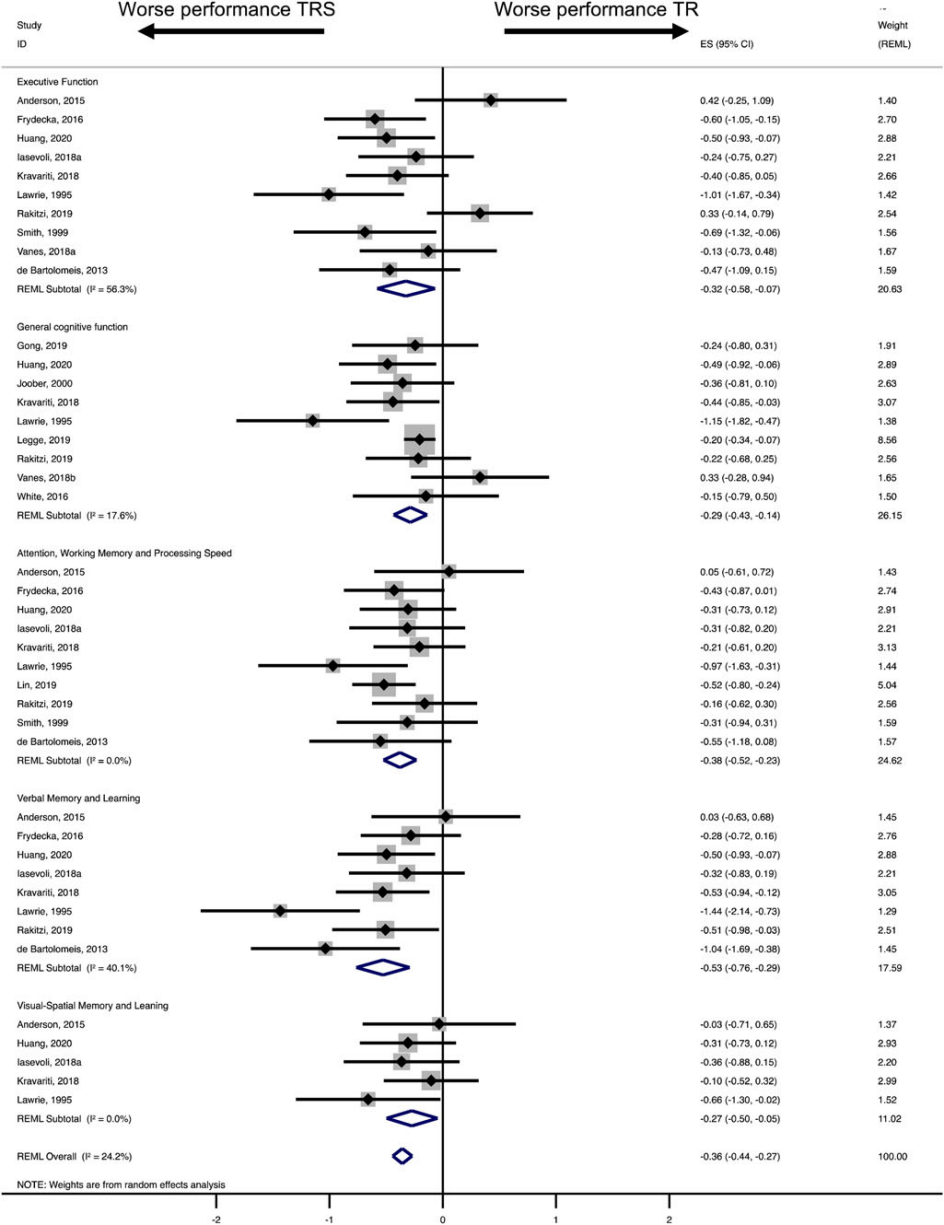
Main analysis: Forest plots of effect sizes of performance differences between treatment-responsive and treatment-resistant patients in 1. executive function, 2. general cognitive function, 3. attention, working memory and processing speed, 4. verbal memory and learning and 5. visual−spatial memory and learning.

Figure 2 shows the forest plots for the five primary meta-analyses. Negative effect sizes (left) indicate worse cognitive performance in treatment-resistant cases. The diamond line shows the overall effect size for each meta-analysis.

### Heterogeneity

Cochrane's *Q* statistic was significant for ‘Executive function’, indicating some degree of heterogeneity (Table 3). Using the *I*^2^ statistic, there was no evidence of heterogeneity (*I*^2^ = 0%) for ‘Attention, working memory and processing speed’ and ‘Visual−spatial memory’, and small heterogeneity (*I*^2^ = 17.59%) for ‘General cognitive function’ (Table 3). However, there was moderate heterogeneity (defined as ‘30–60%’; Ryan, 2016) for ‘Executive function’ and ‘Verbal memory and learning’ (Table 3). Meta-regressions showed no significant effects of demographic, clinical and medication variables on the overall effect sizes of ‘Executive function’ and ‘Verbal memory and learning’ (online Supplementary Table S5).

### Publication bias

**Fig. 3.**
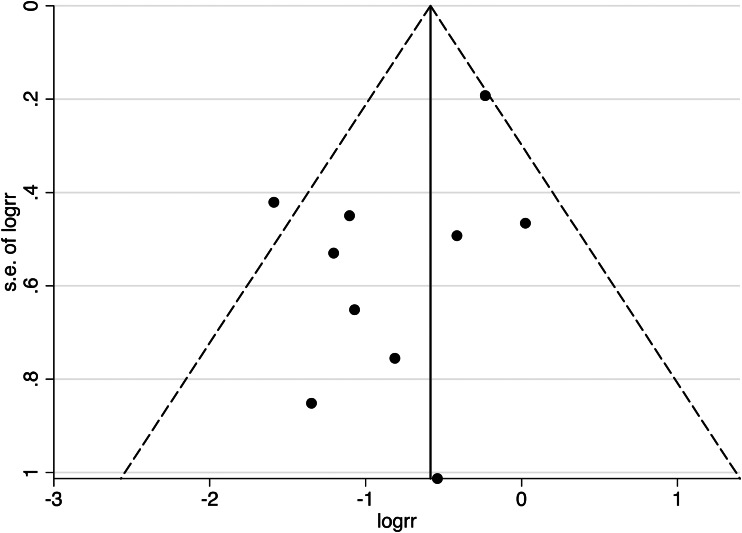
A funnel plot for all 17 publications was included in the main analysis, with 95% confidence interval limits. Data points at the top of the funnel originate from larger sampled investigations.

To assess the potential of publication bias, a funnel plot for all datapoints across cognitive domains was generated and examined using visual inspection and Egger's test (Egger, Smith, Schneider, & Minder, 1997). The latter can help detect bias with a smaller number of publications (Egger et al., 1997). The funnel plot was symmetrical (Fig. 3) and the Egger's test indicated no presence of publication bias (*t*(10) = −1.80, *p* = 0.109).

### Quality of studies

All publications received ‘*good’* (*N* = 12) or ‘*fair’* (*N* = 5) ratings following quality assessments (online Supplementary Table S3), indicating no bias due to flaws in study design or implementation, or some bias, but not sufficient to invalidate the study results (NIH, 2014b).

### Sub-analysis: Meta-analysis of language-related functions from across primary cognitive domains

Table 3 illustrates the REML findings and heterogeneity estimates for the main analysis (five primary cognitive domains) and sub-analysis (language-related functions). The forest plot of the sub-analysis is further presented in online Supplementary Fig. S1. The meta-analysis of language-related functions gave rise to a moderate effect size, closely comparable to the effect size for verbal memory and learning (*dl* = −0.53, 95% CI −0.82 to −0.23; *z* = 3.45; *p* < 0.001) (Table 3, online Supplementary Fig. S1). The Cochrane's *Q* test (*Q* = 13.96; *p* = 0.016) and the *I*^2^ index (64.17%) suggested substantial heterogeneity in effect sizes across studies (Ryan, 2016). Meta-regressions showed no significant effects of demographic, clinical or medication variables on effect sizes (online Supplementary Table S5).

### Sensitivity analysis: Clozapine response

Online Supplementary Figs S2 and S3 present the results of the meta-analyses for the five cognitive domains after excluding the clozapine-responsive TRS samples from Anderson et al. (2015) and Lin et al. (2019) (online Supplementary Fig. S2) and after adding the clozapine-resistant TRS samples from both studies (Anderson et al., 2015; Lin et al., 2019) (online Supplementary Fig. S3). Manipulating the sampling strategy in this way did not alter the pattern of findings from that reported for the main analysis (online Supplementary Figs S2 and S3).

## Discussion

This is the first systematic review and meta-analysis to compare neuropsychological performance between treatment-resistant (*n* = 925) and treatment-responsive (*n* = 939) patients with predominantly chronic schizophrenia across eligible published studies (*n* = 17). Meta-analyses were performed in relation to five cognitive domains, including executive function, general cognitive function, attention, working memory and processing speed, verbal memory and learning, and visual−spatial memory and learning. As part of a focused sub-analysis, we further meta-analysed findings in relation to variables with a prominent language processing component from across the primary cognitive domains. We finally performed separate sensitivity analyses to examine the effect of clozapine response on the main findings.

Confirming our hypotheses, all meta-analyses generated small to moderate effect sizes, which were statistically significant for all but two domains (executive function; visual−spatial memory and learning) and most pronounced for verbal memory and learning and language-related functions. These results suggest that chronic patients with TRS show wide-ranging neuropsychological deficits compared to those with treatment-responsive schizophrenia, which are most salient in verbal functions. Manipulating our sampling strategy to include or exclude samples selected for clozapine response did not affect the main pattern of findings.

### Salience of verbal memory deficits in TRS

Verbal memory and learning consistently show the largest effect sizes in meta-analyses of neuropsychological deficits in first-episode (Mesholam-Gately, Giuliano, Goff, Faraone, & Seidman, 2009), drug-naïve (Fatouros-Bergman et al., 2014) and chronic (Heinrichs & Zakzanis, 1998) schizophrenia patients compared to healthy controls. The impairment is not secondary to IQ deficits (Kravariti et al., 2009) and is also seen in an attenuated form in unaffected first-degree relatives of schizophrenia patients (Bora, Akdede, & Alptekin, 2017; Snitz, MacDonald, & Carter, 2006). This empirical research suggests that verbal memory impairment is an endophenotype for schizophrenia (McCarthy et al., 2018) and taps into core pathophysiological processes in the disorder (Kravariti et al., 2009).

Confirming our hypothesis and extending earlier findings (Fatouros-Bergman et al., 2014; Heinrichs & Zakzanis, 1998; Mesholam-Gately et al., 2009), verbal memory and learning emerged as one of two cognitive aspects best discriminating between treatment-resistant and treatment-responsive patients in the present meta-analysis.

### Salience of language function deficits in TRS

Language functions have been reported to distinguish between TRS patients and treatment-responsive controls already at the first episode (Kravariti et al., 2018). Our sub-analysis of language-related functions gave rise to an effect size comparable to that detected for verbal memory and learning in the main analysis. Early emergence of language-related deficits in TRS patients compared to treatment responders might reflect a greater contribution of neurodevelopmental impairment in the former group.

Interestingly, verbal intelligence and language deficits are among the less distinctive features of the neuropsychological profile of schizophrenia at large (Kravariti et al., 2009; Mesholam-Gately et al., 2009). For example, language functions were only the fifth most impaired domain in a meta-analysis of neuropsychological deficits in first-episode schizophrenia patients compared to healthy controls (Mesholam-Gately et al., 2009), while premorbid verbal and non-verbal intelligence are equally impaired in population-based studies (Khandaker, Barnett, White, & Jones, 2011). A differential salience of language function deficits in the comparative neuropsychological profiles of TRS individuals and of schizophrenia patients at large might be underpinned by a qualitative neuropsychological difference between treatment-resistant and treatment-responsive schizophrenia. If confirmed, such distinction will be of great theoretical and practical interest, for example, in developing aetiological models and personalised medicine in TRS.

### Origins of neuropsychological deficits in TRS

The effect sizes that emerged in our meta-analyses (0.27–0.53) are of similar magnitude to those reported in meta-analyses of neuropsychological findings from unaffected first-degree relatives of schizophrenia patients relative to healthy controls (0.20–0.66) (Bora et al., 2017; Snitz et al., 2006). Combined, the above findings raise the possibility of a genetic and cognitive continuum of schizophrenia risk, which increases from undiagnosed community controls to unaffected first-degree relatives of schizophrenia patients to treatment-responsive schizophrenia patients to TRS patients.

A broader hypothesis is that TRS is aetiologically continuous with treatment-responsive schizophrenia but occupies a more extreme position in a continuum of neurodevelopmental liability. This hypothesis is in keeping with findings relating to predictors of poor therapeutic response, which largely coincide with the defining features of neurodevelopmental schizophrenia (Carbon & Correll, 2014; Murray et al., 1992).

The dearth of neuropsychological investigations into the first psychotic episode of patients who develop TRS limits inferences on the origin and stability of the neuropsychological gradient between TRS and treatment-responsive schizophrenia. Based on limited findings to date, this differential is likely to predate clinical onset in relation to language functions (Kravariti et al., 2018).

### Methodological considerations

This is the first systematic review and meta-analysis of neuropsychological deficits in treatment-resistant, relative to treatment-responsive, schizophrenia. The originality of our research undertaking, our systematic methodological approach, and the fair/good quality of the original studies are strengths of the present investigation.

Integrated with earlier research, our findings offer new insights into possible aetiological contributions to TRS, but they need to be viewed in the light of some limitations: Even though the largest effects sizes for verbal memory and learning and for language-related functions were moderate, thus distinguishable from the remaining (small) effect sizes, there was substantial overlap in 95% CIs. This might suggest that the true mean differences between the schizophrenia populations of interest might be less pronounced than our estimates suggest. Our conservative sampling and analytic strategies (biasing estimates towards conservative rather than inflated estimates) are likely to have mitigated this risk.

Our heterogeneity analyses suggested moderate-to-substantial inconsistency of effect sizes for executive function, verbal memory and learning, and language-related functions. This inconsistency was statistically addressed by employing random-effects models (Langan et al., 2019; Tanriver-Ayder, Faes, van de Casteele, McCann, & Macleod, 2021; Veroniki et al., 2019), and, where appropriate, by performing meta-regressions (Ryan, 2016). However, the relatively small number of studies (*n* = 5–10) prevented subgroup analyses and is likely to also explain the lack of statistically significant findings in our meta-regressions.

Traditional conceptualisations of verbal fluency (VF) see VF as primarily an ‘executive function’ in the literature (Henry & Crawford, 2005; Joyce, Collinson, & Crichton, 1996). However, more recent factor-analytic evidence suggests that both letter (phonemic) and category (semantic) fluency are more closely related to language than to executive function (Whiteside et al., 2016). We addressed this duality by classifying verbal fluency as an executive function in our main analysis and as a language-related function in our sub-analysis.

Most neuropsychological studies of TRS to date have been cross-sectional and included chronic patient samples, which has limited the scope of the present meta-analysis. In the absence of first-episode studies and longitudinal designs, it is difficult to distinguish between neuropsychological deficits that tap into the core pathophysiology of TRS from those secondary to the combined effects of chronicity and persistent poor regulation of clinical symptoms.

A noteworthy limitation of our meta-analysis is the scarcity of publications that differentiated between clozapine-responsive and clozapine-resistant TRS subgroups. The dearth of relevant studies prevented us from addressing the critical importance of clozapine response (Farooq et al., 2019; Lindenmayer et al., 2019; Moulier et al., 2021; Potkin et al., 2020; Siskind et al., 2017) in subgroup analyses. Although our sensitivity analysis produced nearly identical results to those of the main analysis, this is a likely reflection of the small number of studies underpinning it.

## Conclusions and future directions

Patients with TRS show wide-ranging deficits of small to moderate effect sizes compared to treatment responders, which are most salient in verbal memory and learning and in language functions. The latter is of particular interest to theoretical and research explorations of treatment resistance, as they are likely markers of neurodevelopmental vulnerability to TRS.

A hypothesis that merits exploration in future research is that core deficits in language functions, a neurodevelopmental aetiology, and a primary glutamatergic dysfunction converge into a single model of TRS. In support of a model which helps to bridge glutamate and neurodevelopmental hypotheses of TRS, glutamate is associated with schizophrenia in genetic association analyses (Spangaro et al., 2012), with verbal fluency deficits in high-risk individuals (Allen et al., 2015) and, critically, with several language-related neurodevelopmental processes (Lebel, MacMaster, & Dewey, 2016; Takenouchi et al., 2014).

Future studies should employ longitudinal controlled designs extending from the high-risk to the first episode and onto the chronic stages of TRS, as well as undertake incisive comparisons across treatment-responsive, clozapine-responsive and clozapine-resistant subgroups of schizophrenia patients. Digit Symbol would be an interesting focus of future meta-analyses in TRS. The task taps into a distinct, marked and neurobiologically significant impairment, which exceeds that of other traditional neuropsychological tasks (Dickinson, Ramsey, & Gold, 2007). The ultimate goal of the neuropsychological characterisation of TRS is to help advance the translational scope of research into TRS, particularly in relation to personalised medicine. Recent findings have stirred optimism in this direction. For example, a reported significant alteration in cognitive flexibility in TRS is believed to tap into a distinct underlying neurobiological mechanism and may inform future treatment strategies (e.g. glutamatergic targets and giving clozapine earlier in resistant patients) (Horne et al., 2021). Our findings suggest that language-related tasks, and potentially verbal memory and learning tasks, have likely applications in multidisciplinary strategies to elucidating the pathophysiology of TRS and to developing predictive models and personalised medical approaches.

## Supplementary material

For supplementary material accompanying this paper visit https://doi.org/10.1017/S0033291721004128. click here to view supplementary material
