# Supplementary material for: Neuropsychological differences between treatment-resistant and treatment-responsive schizophrenia: a meta-analysis
Source: Psychol Med. 2021 Nov 1;52(1):1–13. doi: 10.1017/S0033291721004128 (PMC8711103; doi:10.1017/S0033291721004128)
Supplement: Supplementary file 1 [file S0033291721004128sup001.docx]

**Supplementary material**

**Table S.1……** PRISMA checklist (from Moher, Liberati, Tetzlaff & Altman, 2009 and Liberati et al., 2009)

**Table S.2……** Diagnostic criteria for schizophrenia spectrum disorders and definition of treatment-resistant and treatment-responsive schizophrenia in the 17 publications

**Table S.3……** Overall quality ratings of the 17 publications

**Table S.4……** Means and standard deviations of cognitive tasks in treatment-resistant and treatment-responsive patients in the 17 publications

**Table S.5……** Meta-regression analysis for cognitive domains that showed moderate to substantial heterogeneity of effect sizes in the main analysis and sub-analysis

**Figure S.1……** Forest plots of effect sizes of performance differences in language-related functions between treatment-responsive and treatment-resistant patients (sub-analysis)

**Figure S.2……** Sensitivity analysis: Forest plot of effect sizes of performance differences between treatment-resistant and treatment-responsive schizophrenia patients after excluding clozapine-responsive TRS samples (Anderson et al., 2015; Lin et al., 2019)

**Figure S.3……** Sensitivity analysis: Forest plot of effect sizes of performance differences between treatment-resistant and treatment-responsive schizophrenia patients after adding^1^ clozapine-resistant TRS samples (Anderson et al., 2015; Lin et al., 2019)

**Table S.1**

*PRISMA checklist (from Moher, Liberati, Tetzlaff & Altman, 2009 and Liberati et al., 2009)*

| **Section/topic** | **#** | **Checklist item** | **Reported on page #** |
| --- | --- | --- | --- |
| **TITLE** | | |  |
| Title | 1 | Identify the report as a systematic review, meta-analysis, or both. | 1 |
| **ABSTRACT** | | |  |
| Structured summary | 2 | Provide a structured summary including, as applicable: background; objectives; data sources; study eligibility criteria, participants, and interventions; study appraisal and synthesis methods; results; limitations; conclusions and implications of key findings; systematic review registration number. | 2 |
| **INTRODUCTION** | | |  |
| Rationale | 3 | Describe the rationale for the review in the context of what is already known. | 3/4 |
| Objectives | 4 | Provide an explicit statement of questions being addressed with reference to participants, interventions, comparisons, outcomes, and study design (PICOS). | 4/5 |
| **METHODS** | | |  |
| Protocol and registration | 5 | Indicate if a review protocol exists, if and where it can be accessed (e.g., Web address), and, if available, provide registration information including registration number. | 4/5 |
| Eligibility criteria | 6 | Specify study characteristics (e.g., PICOS, length of follow-up) and report characteristics (e.g., years considered, language, publication status) used as criteria for eligibility, giving rationale. | 5 |
| Information sources | 7 | Describe all information sources (e.g., databases with dates of coverage, contact with study authors to identify additional studies) in the search and date last searched. | 4/5 |
| Search | 8 | Present full electronic search strategy for at least one database, including any limits used, such that it could be repeated. | 4/5/Figure 1 |
| Study selection | 9 | State the process for selecting studies (i.e., screening, eligibility, included in systematic review, and, if applicable, included in the meta-analysis). | 5/Figure 1 |
| Data collection process | 10 | Describe method of data extraction from reports (e.g., piloted forms, independently, in duplicate) and any processes for obtaining and confirming data from investigators. | 5/6/Figure 1 |
| Data items | 11 | List and define all variables for which data were sought (e.g., PICOS, funding sources) and any assumptions and simplifications made. | 5/6 |
| Risk of bias in individual studies | 12 | Describe methods used for assessing risk of bias of individual studies (including specification of whether this was done at the study or outcome level), and how this information is to be used in any data synthesis. | 6/7 |
| Summary measures | 13 | State the principal summary measures (e.g., risk ratio, difference in means). | 6 |
| Synthesis of results | 14 | Describe the methods of handling data and combining results of studies, if done, including measures of consistency (e.g., I^2^) for each meta-analysis. | 6/7 |

| **Section/topic** | **#** | **Checklist item** | **Reported on page #** |
| --- | --- | --- | --- |
| Risk of bias across studies | 15 | Specify any assessment of risk of bias that may affect the cumulative evidence (e.g., publication bias, selective reporting within studies). | 6/7/Supplementary |
| Additional analyses | 16 | Describe methods of additional analyses (e.g., sensitivity or subgroup analyses, meta-regression), if done, indicating which were pre-specified. | 8/Supplementary |
| **RESULTS** | | |  |
| Study selection | 17 | Give numbers of studies screened, assessed for eligibility, and included in the review, with reasons for exclusions at each stage, ideally with a flow diagram. | 5/Figure 1 |
| Study characteristics | 18 | For each study, present characteristics for which data were extracted (e.g., study size, PICOS, follow-up period) and provide the citations. | 6/7/8/Table 2 |
| Risk of bias within studies | 19 | Present data on risk of bias of each study and, if available, any outcome level assessment (see item 12). | 10/11/Table 3/Supplementary |
| Results of individual studies | 20 | For all outcomes considered (benefits or harms), present, for each study: (a) simple summary data for each intervention group (b) effect estimates and confidence intervals, ideally with a forest plot. | 9/10/Table 3/ Figure 2 |
| Synthesis of results | 21 | Present results of each meta-analysis done, including confidence intervals and measures of consistency. | 9/10/Table 3/Figure 2 |
| Risk of bias across studies | 22 | Present results of any assessment of risk of bias across studies (see Item 15). | 9/10/11/Figure 3/Supplementary |
| Additional analysis | 23 | Give results of additional analyses, if done (e.g., sensitivity or subgroup analyses, meta-regression [see Item 16]). | 11/Supplementary |
| **DISCUSSION** | | |  |
| Summary of evidence | 24 | Summarize the main findings including the strength of evidence for each main outcome; consider their relevance to key groups (e.g., healthcare providers, users, and policy makers). | 12/13 |
| Limitations | 25 | Discuss limitations at study and outcome level (e.g., risk of bias), and at review-level (e.g., incomplete retrieval of identified research, reporting bias). | 14/15 |
| Conclusions | 26 | Provide a general interpretation of the results in the context of other evidence, and implications for future research. | 14/15 |
| **FUNDING** | | |  |
| Funding | 27 | Describe sources of funding for the systematic review and other support (e.g., supply of data); role of funders for the systematic review. | 16 |

**Table S.2**

*Diagnostic criteria for schizophrenia spectrum disorders and definition of treatment-resistant and treatment-responsive schizophrenia in the 17 publications*

| Publication (year) | Diagnostic criteria: Psychosis/Schizophrenia | Definition of treatment resistant schizophrenia (TRS) | Definition of treatment responsive schizophrenia |
| --- | --- | --- | --- |
| Anderson et al (2015) | DSM-IV criteria for schizophrenia | Failure to respond (as indicated by clinical ratings of symptom severity) to at least two or more antipsychotic trials at adequate duration (4 - 6 weeks) within an acceptable therapeutic dose | Evidence of remission or stabilisation of symptoms following first-line antipsychotic medication |
| Bourque et al (2013) | DSM-IV criteria for schizophrenia | Treatment with clozapine due to resistance to previous pharmacological treatments | Evidence of remission or stabilisation of symptoms following antipsychotic medication |
| de Bartolomeis et al (2013) | DSM-IV criteria for schizophrenia | Failure to respond (as indicated by clinical ratings of symptom severity) to at least two or more antipsychotic trials at adequate duration (4 - 6 weeks) within an acceptable therapeutic dose. | Not reaching criteria for treatment resistance (TRS) |
| Frydecka et al (2016) | DSM-IV & ICD-10 criteria for schizophrenia | Failure to respond (as indicated by clinical ratings of symptom severity) to at least two or more antipsychotic trials at adequate duration (4 - 6 weeks) within an acceptable therapeutic dose. | N/R |
| Gong et al (2019) | DSM-IV & DSM-V criteria for schizophrenia | Failure to respond (as indicated by clinical ratings of symptom severity) to at least two or more antipsychotic trials at adequate duration (4 - 6 weeks) within an acceptable therapeutic dose | N/R |
| Huang et al (2020) | DSM-IV criteria for schizophrenia | Failure to respond (as indicated by clinical ratings of symptom severity) to at least two or more antipsychotic trials at adequate duration (4 - 6 weeks) within an acceptable therapeutic dose. | Evidence of remission or stabilisation of symptoms following antipsychotic medication |
| Iasevoli et al (2018a) | DSM-IV criteria for schizophrenia | Failure to respond (as indicated by clinical ratings of symptom severity) to at least two or more antipsychotic trials at adequate duration (4 - 6 weeks) within an acceptable therapeutic dose. | Evidence of remission or stabilisation of symptoms following antipsychotic medication |
| Joober et al (2002) | DSM-IV criteria for schizophrenia | Failure to respond (as indicated by clinical ratings of symptom severity) to at least two or more antipsychotic trials at adequate duration (4 - 6 weeks) within an acceptable therapeutic dose. | Evidence of remission or stabilisation of symptoms following antipsychotic medication |
| Kravariti et al (2018) | ICD-10 criteria for schizophrenia | Failure to respond (as indicated by clinical ratings of symptom severity) to at least two or more antipsychotic trials at adequate duration (4 - 6 weeks) within an acceptable therapeutic dose. | Score of 2 or less on the Schedules for Clinical Assessment in Neuropsychiatry (SCAN; WHO, 1992), with this stable for at least 6 months (Andreasen et al., 2005) |
| Lawrie et al (1995) | DSM-III-R criteria for schizophrenia | Criteria proposed by May et al 1988 (see Brenner et al., 1990) which describes seven levels of treatment refraction. Level five or above: moderately persistent symptoms in personal and social adjustment, following at least three or more antipsychotic trials at adequate duration (6 weeks) within an acceptable therapeutic dose. | Level three of below (see Brenner et al., 1990): evidence of slow or rapid response to antipsychotic medication with only residual symptoms present |
| Legge et al (2019) | DSM-IV & ICD-10 criteria for schizophrenia | Treatment with clozapine or rated negatively for OPCRIT item 89 (*psychotic symptoms respond to neuroleptics*) | Rated positively for OPCRIT item 89 (*psychotic symptoms respond to neuroleptics*) and never treated with clozapine |
| Lin et al (2019) | DSM-IV criteria for schizophrenia | Failure to respond (as indicated by clinical ratings of symptom severity) to at least two or more antipsychotic trials at adequate duration (4 - 6 weeks) within an acceptable therapeutic dose. | Not reaching criteria for treatment resistance (TRS) |
| Rakitzi et al (2019) | DSM-IV criteria for schizophrenia | Failure to respond (as indicated by clinical ratings of symptom severity) to at least two or more antipsychotic trials at adequate duration (4 - 6 weeks) within an acceptable therapeutic dose. | N/R |
| Smith et al (1999) | DSM-III-R criteria for schizophrenia | Failure to respond (as indicated by clinical ratings of symptom severity) to at least two or more antipsychotic trials at adequate duration (six weeks) within an acceptable therapeutic dose. | Evidence of remission or stabilisation of symptoms following antipsychotic medication |
| Vanes et al (2018a) | ICD-10 criteria for schizophrenia | Failure to respond (as indicated by clinical ratings of symptom severity) to at least two or more antipsychotic trials at adequate duration (4 - 6 weeks) within an acceptable therapeutic dose. | Score of 3 or less on all PANSS items (Conley and Kelly, 2001), with this stable for at least 6 months (Andreasen et al., 2005) |
| Vanes et al (2018b) | ICD-10 criteria for schizophrenia | Failure to respond (as indicated by clinical ratings of symptom severity) to at least two or more antipsychotic trials at adequate duration (4 - 6 weeks) within an acceptable therapeutic dose. | Score of 3 or less on all PANSS items (Conley and Kelly, 2001), with this stable for at least 6 months (Andreasen et al., 2005) |
| White et al (2016) | DSM-IV criteria for schizophrenia | Failure to respond (as indicated by clinical ratings of symptom severity) to at least two or more antipsychotic trials at adequate duration (4 - 6 weeks) within an acceptable therapeutic dose. | Not reaching criteria for treatment resistance (TRS) |

*Note:*  N/R = not reported.

**Table S.3**

*Overall quality ratings of the 17 publications*

| Publication | Design | Quality appraisal scale | Quality appraisal (Good, Fair, Poor) |
| --- | --- | --- | --- |
| Anderson et al., 2015 | Cross-sectional | NHLBI: Quality Assessment Tool for Observational Cohort and Cross-Sectional Studies | Good |
| Bourque et al., 2013 | Cross-sectional | NHLBI: Quality Assessment Tool for Observational Cohort and Cross-Sectional Studies | Fair |
| de Bartolomeis et al., 2013 | Cross-sectional | NHLBI: Quality Assessment Tool for Observational Cohort and Cross-Sectional Studies | Good |
| Frydecka et al., 2016 | Cross-sectional | NHLBI: Quality Assessment Tool for Observational Cohort and Cross-Sectional Studies | Good |
| Gong et al., 2019 | Cross-sectional | NHLBI: Quality Assessment Tool for Observational Cohort and Cross-Sectional Studies | Good |
| Huang et al., 2020 | Cross-sectional | NHLBI: Quality Assessment Tool for Observational Cohort and Cross-Sectional Studies | Good |
| Iasevoli et al., 2018a | Cross-sectional | NHLBI: Quality Assessment Tool for Observational Cohort and Cross-Sectional Studies | Good |
| Joober et al., 2002 | Cross-sectional | NHLBI: Quality Assessment Tool for Observational Cohort and Cross-Sectional Studies | Fair |
| Kravariti et al., 2018 | Cross-sectional | NHLBI: Quality Assessment Tool for Observational Cohort and Cross-Sectional Studies | Good |
| Lawrie et al., 1995 | Cross-sectional | NHLBI: Quality Assessment Tool for Observational Cohort and Cross-Sectional Studies | Fair |
| Legge et al., 2019 | Cross-sectional | NHLBI: Quality Assessment Tool for Observational Cohort and Cross-Sectional Studies | Good |
| Lin et al., 2019 | Cross-sectional | NHLBI: Quality Assessment Tool for Observational Cohort and Cross-Sectional Studies | Good |
| Rakitzi & Georgila, 2019 | Randomized Control Trial | NHLBI: Quality Assessment for Controlled Intervention Studies | Good |
| Smith, Kadewari, Rosenberger & Bhattacharyya, 1999 | Cross-sectional | NHLBI: Quality Assessment Tool for Observational Cohort and Cross-Sectional Studies | Fair |
| Vanes et al., 2018a | Cross-sectional | NHLBI: Quality Assessment Tool for Observational Cohort and Cross-Sectional Studies | Good |
| Vanes et al., 2018b | Cross-sectional | NHLBI: Quality Assessment Tool for Observational Cohort and Cross-Sectional Studies | Fair |
| White et al., 2019 | Cross-sectional | NHLBI: Quality Assessment Tool for Observational Cohort and Cross-Sectional Studies | Good |

**Table S.4**

*Means and standard deviations of cognitive tasks in treatment-resistant and treatment-responsive patients in the 17 publications*

|  |  |  |  |  | Treatment responsive |  |  | Treatment resistant (TRS) |  |
| --- | --- | --- | --- | --- | --- | --- | --- | --- | --- |
| Cognitive domain | Publication | Cognitive task (subtest) | Sample size | N | Mean | SD | N | Mean | SD |
| **Main Analysis ^1^** | | | | | | | | | |
| Executive function  (10 publications, 24 comparisons in cognitive performance between treatment-responsive and TRS participants) | de Bartolomeis et al., 2013 | BACS Tower of London | 41 | 22 | 1.36 | 1.50 | 19 | .76 | 1.08 |
|  | de Bartolomeis et al., 2013 | BACS Category Instances Task | 41 | 22 | 1.32 | 1.20 | 19 | .78 | .91 |
|  | Anderson et al., 2015 | BRCCB Information Processing Speed | 36 | 16 | -1.06 | 1.27 | 20 | -0.99 | .73 |
|  | Anderson et al., 2015 | BRCCB Information Processing Efficiency | 36 | 16 | -0.74 | .98 | 20 | -0.54 | .98 |
|  | Anderson et al., 2015 | BRCCB Verbal Fluency | 36 | 16 | -1.05 | .77 | 20 | -0.54 | .98 |
|  | Frydecka et al., 2016 | Trail Making B* | 85 | 32 | 99.70 | 63.21 | 53 | 140.56 | 93.64 |
|  | Frydecka et al., 2016 | Stroop Test (incongruent trial)* | 85 | 32 | 62.80 | 24.90 | 53 | 85.00 | 28.07 |
|  | Frydecka et al., 2016 | Semantic Verbal Fluency | 85 | 32 | 18.67 | 7.43 | 53 | 15.33 | 5.45 |
|  | Frydecka et al., 2016 | Phonological Verbal Fluency | 85 | 32 | 26.80 | 11.59 | 53 | 21.47 | 7.69 |
|  | Huang et al., 2020 | Neuropsychological Assessment Battery: Mazes | 86 | 43 | 50.60 | 11.30 | 43 | 44.90 | 11.40 |
|  | Iasevoli et al., 2018a | BACS Tower of London | 60 | 32 | 1.68 | 1.32 | 28 | 1.11 | 1.48 |
|  | Iasevoli et al., 2018a | BACS Category Instances Task | 60 | 32 | 1.00 | 1.27 | 28 | .91 | 1.29 |
|  | Kravariti et al., 2018 | Trail Making B* | 135 | 108 | 96.15 | 57.66 | 27 | 127.04 | 122.63 |
|  | Kravariti et al., 2018 | Letter-Number Span | 133 | 105 | 13.10 | 3.97 | 28 | 11.96 | 4.77 |
|  | Kravariti et al., 2018 | Semantic Verbal Fluency | 115 | 91 | 35.16 | 9.34 | 24 | 31.50 | 1.29 |
|  | Kravariti et al., 2018 | Phonological Verbal Fluency | 114 | 90 | 22.71 | 9.12 | 24 | 18.96 | 9.48 |
|  | Lawrie et al., 1995 | Stroop Test (incongruent trial: colour error)* | 40 | 20 | 16.70 | 17.90 | 20 | 41.00 | 19.50 |
|  | Lawrie et al., 1995 | Phonological Verbal Fluency | 40 | 20 | 20.20 | 8.70 | 20 | 14.20 | 7.10 |
|  | Rakitzi & Georgila, 2019 | Letter-Number Span | 72 | 39 | 12.50 | 3.71 | 33 | 13.93 | 4.94 |
|  | Smith et al., 1999 | Trail Making B (log sec)* | 39 | 16 | 2.16 | .21 | 23 | 2.28 | .29 |
|  | Smith et al., 1999 | Modified Wisconsin Card Sorting Test (Perseverative Errors %)* | 45 | 20 | 65.80 | 31.80 | 25 | 81.10 | 30.00 |
|  | Smith et al., 1999 | Modified Wisconsin Card Sorting Test (Total errors)* | 45 | 20 | 29.70 | 12.20 | 25 | 38.40 | 6.20 |
|  | Smith et al., 1999 | Phonological and Semantic Verbal Fluency | 43 | 19 | 63.40 | 18.00 | 24 | 38.90 | 24.10 |
|  | Vanes et al., 2018a | Stroop Test (incongruent trial: stroop effect)* | 42 | 21 | 169.78 | 129.95 | 21 | 186.66 | 128.26 |
| General cognitive functioning  (9 publications, 12 comparisons in cognitive performance between treatment-responsive and TRS participants) | Gong et al., 2019 | Mini Mental State Examination | 53 | 20 | 24.10 | 9.60 | 33 | 21.50 | 11.00 |
|  | Huang et al., 2020 | MCCB Composite | 86 | 43 | 45.40 | 10.60 | 43 | 40.40 | 9.70 |
|  | Joober et al., 2002 | Mini Mental State Examination | 75 | 36 | 32.30 | 1.97 | 39 | 31.40 | 2.91 |
|  | Kravariti et al., 2018 | WAIS-R Full Scale IQ | 139 | 109 | 90.82 | 14.92 | 30 | 81.83 | 14.84 |
|  | Kravariti et al., 2018 | National Adult Reading Test | 135 | 106 | 98.88 | 14.67 | 29 | 94.86 | 12.79 |
|  | Lawrie et al., 1995 | Mini Mental State Examination | 40 | 20 | 28.30 | 1.80 | 20 | 25.20 | 3.70 |
|  | Lawrie et al., 1995 | Quick Test IQ | 40 | 20 | 107.40 | 18.00 | 20 | 89.10 | 14.90 |
|  | Lawrie et al., 1995 | National Adult Reading Test | 40 | 20 | 114.5 | 10.3 | 20 | 101.2 | 9.5 |
|  | Legge et al., 2019 | National Adult Reading Test | 817 | 361 | 99.53 | 13.34 | 456 | 96.78 | 13.54 |
|  | Rakitzi & Georgila, 2019 | WAIS-R Full Scale IQ | 72 | 39 | 90.61 | 8.35 | 33 | 88.75 | 8.68 |
|  | Vanes et al., 2018b | WASI Full Scale IQ (two-subtests) | 42 | 21 | 91.86 | 14.80 | 21 | 97.10 | 16.40 |
|  | White et al., 2016 | WAIS-R Full Scale IQ | 38 | 22 | 99.09 | 12.57 | 16 | 96.81 | 17.82 |
| Attention, working memory and processing speed  (10 publications, 24 comparisons in cognitive performance between treatment-responsive and TRS participants) | de Bartolomeis et al., 2013 | BACS Digit Sequencing Task | 41 | 22 | 1.59 | 1.35 | 19 | .92 | 1.48 |
|  | de Bartolomeis et al., 2013 | BACS Symbol Coding Task | 41 | 22 | .69 | 1.06 | 19 | .16 | .39 |
|  | Anderson et al., 2015 | BRCCB Working Memory | 36 | 16 | -0.55 | .83 | 20 | -0.74 | .81 |
|  | Anderson et al., 2015 | BRCCB Sustained Attention | 36 | 16 | -1.11 | 1.18 | 20 | -0.68 | 1.30 |
|  | Frydecka et al., 2016 | Trail Making A* | 85 | 32 | 38.53 | 17.25 | 53 | 48.45 | 20.73 |
|  | Frydecka et al., 2016 | Stroop Test (congruent trial)* | 85 | 32 | 36.73 | 6.94 | 53 | 50.22 | 34.09 |
|  | Frydecka et al., 2016 | WAIS-R Digit Symbol Coding Test | 85 | 32 | 43.07 | 13.28 | 53 | 34.10 | 13.63 |
|  | Frydecka et al., 2016 | WAIS-R Digit Span Forward | 85 | 32 | 6.30 | 1.99 | 53 | 6.16 | 1.71 |
|  | Frydecka et al., 2016 | WAIS-R Digit Span Backward | 85 | 32 | 5.72 | 2.28 | 53 | 4.90 | 1.69 |
|  | Huang et al., 2020 | MCCB Attention/Vigilance | 86 | 43 | 46.00 | 9.60 | 43 | 45.20 | 9.80 |
|  | Huang et al., 2020 | MCCB Working Memory | 86 | 43 | 47.50 | 10.00 | 43 | 41.20 | 13.20 |
|  | Iasevoli et al., 2018a | Continuous Performance Test | 60 | 32 | 36.66 | 17.71 | 28 | 31.99 | 17.89 |
|  | Iasevoli et al., 2018a | BACS Digit Sequencing Task | 60 | 32 | 1.19 | 1.31 | 28 | .66 | .74 |
|  | Iasevoli et al., 2018a | BACS Symbol Coding Task | 60 | 32 | .56 | .87 | 28 | .39 | .81 |
|  | Kravariti et al., 2018 | Trail Making A* | 136 | 106 | 45.42 | 27.22 | 30 | 51.07 | 32.75 |
|  | Kravariti et al., 2018 | WAIS-R Digit Symbol Coding Test | 136 | 106 | 7.14 | 2.60 | 30 | 6.57 | 2.75 |
|  | Lawrie et al., 1995 | WAIS-R Digit Symbol Coding Test | 40 | 20 | 8.60 | 2.40 | 20 | 6.40 | 2.00 |
|  | Lawrie et al., 1995 | WAIS-R Digit Span Backward | 40 | 20 | 5.30 | 1.90 | 20 | 3.70 | 1.30 |
|  | Lawrie et al., 1995 | WAIS-R Digit Span Forward | 40 | 20 | 7.50 | 1.40 | 20 | 7.30 | 1.20 |
|  | Lin et al., 2019 | Continuous Performance Test (undegraded) | 150 | 102 | -2.14 | 1.98 | 48 | -3.31 | 2.62 |
|  | Lin et al., 2019 | Continuous Performance Test (degraded) | 150 | 102 | -1.73 | 1.60 | 48 | -2.65 | 1.80 |
|  | Rakitzi & Georgila, 2019 | Continuous Performance Test (omission errors)* | 72 | 39 | 2.83 | 3.41 | 33 | 3.66 | 6.59 |
|  | Smith et al., 1999 | Trail Making A (log sec)* | 39 | 16 | 1.85 | .22 | 23 | 2.06 | .29 |
|  | Smith et al., 1999 | WAIS-R Digit Span Forward-Backward difference* | 45 | 20 | 3.30 | 1.30 | 25 | 3.20 | .90 |
|  |  |  |  |  |  |  |  |  |  |
| Verbal memory and learning  (8 publications, 12 comparisons in cognitive performance between treatment-responsive and TRS participants) | de Bartolomeis et al., 2013 | BACS List Learning Task | 41 | 22 | 2.19 | 1.44 | 19 | .71 | 1.33 |
|  | Anderson et al., 2015 | BRCCB Verbal Learning and Memory | 36 | 16 | -1.73 | 1.17 | 20 | -1.70 | 1.11 |
|  | Frydecka et al., 2016 | Rey Auditory Verbal Learning Test (immediate recall) | 85 | 32 | 40.14 | 10.76 | 53 | 34.62 | 11.72 |
|  | Frydecka et al., 2016 | Rey Auditory Verbal Learning Test (inference) | 85 | 32 | 8.25 | 2.72 | 53 | 7.08 | 3.19 |
|  | Frydecka et al., 2016 | Rey Auditory Verbal Learning Test (delayed recall) | 85 | 32 | 7.04 | 2.78 | 53 | 5.72 | 3.03 |
|  | Frydecka et al., 2016 | Rey Auditory Verbal Learning Test (recognition) | 85 | 32 | 9.00 | 4.19 | 53 | 9.33 | 4.06 |
|  | Huang et al., 2020 | MCCB Hopkins Verbal Learning Test—Revised | 86 | 43 | 50.60 | 11.30 | 43 | 44.90 | 11.40 |
|  | Iasevoli et al., 2018a | BACS List Learning Task | 60 | 32 | 1.75 | 1.54 | 28 | 1.28 | 1.36 |
|  | Kravariti et al., 2018 | Rey Auditory Verbal Learning Test (immediate recall) | 132 | 102 | 44.61 | 11.69 | 30 | 38.87 | 12.27 |
|  | Kravariti et al., 2018 | Rey Auditory Verbal Learning Test (inference) | 129 | 99 | 9.13 | 3.24 | 30 | 7.17 | 3.72 |
|  | Lawrie et al., 1995 | Rivermead Behavioural Memory Test | 40 | 20 | 21.70 | 1.90 | 20 | 17.30 | 3.80 |
|  | Rakitzi & Georgila, 2019 | Greek Verbal Memory Test | 72 | 39 | 10.83 | 2.64 | 30 | 9.40 | 2.97 |
| Visual-spatial memory and learning  (5 publications, 5 comparisons in cognitive performance between treatment-responsive and TRS participants) | Anderson et al., 2015 | BRCCB Visuospatial Learning and Memory | 34 | 15 | -0.61 | .99 | 15 | -0.65 | 1.43 |
|  | Huang et al., 2020 | MCCB Visual Learning | 86 | 43 | 46.20 | 11.90 | 43 | 42.70 | 10.60 |
|  | Iasevoli et al., 2018a | Brief Visuospatial Memory Test-Revised | 60 | 32 | 33.66 | 13.06 | 28 | 28.28 | 16.17 |
|  | Kravariti et al., 2018 | WAIS-R Visual Reproduction trials | 129 | 101 | 9.51 | 3.23 | 28 | 9.18 | 3.13 |
|  | Lawrie et al., 1995 | CANTAB Spatial Recognition | 40 | 20 | 15.40 | 2.30 | 20 | 13.60 | 15.40 |
| **Sub-Analysis of Language-Related Functions ^2^** | | | | | | | | | |
| Language-related functions  (6 publications, 10 comparisons in cognitive performance between treatment-responsive and TRS participants) | Bourque et al., 2013 | WAIS-III Vocabulary | 43 | 23 | 6.60 | 2.78 | 20 | 5.85 | 2.18 |
|  | Frydecka et al., 2016 | Semantic Verbal Fluency | 85 | 32 | 18.67 | 7.43 | 53 | 15.33 | 5.45 |
|  | Frydecka et al., 2016 | Phonological Verbal Fluency | 85 | 32 | 26.80 | 11.59 | 53 | 21.47 | 7.69 |
|  | Kravariti et al., 2018 | Semantic Verbal Fluency | 115 | 91 | 35.16 | 9.34 | 24 | 31.50 | 1.29 |
|  | Kravariti et al., 2018 | Phonological Verbal Fluency | 114 | 90 | 22.71 | 9.12 | 24 | 18.96 | 9.48 |
|  | Kravariti et al., 2018 | National Adult Reading Test | 135 | 106 | 98.88 | 14.67 | 29 | 94.86 | 12.79 |
|  | Kravariti et al., 2018 | WAIS-III Vocabulary | 139 | 109 | 8.31 | 2.50 | 30 | 6.60 | 2.67 |
|  | Lawrie et al., 1995 | National Adult Reading Test | 40 | 20 | 114.5 | 10.3 | 20 | 101.2 | 9.5 |
|  | Legge et al., 2019 | National Adult Reading Test | 817 | 361 | 99.53 | 13.34 | 456 | 96.78 | 13.54 |
|  | Smith et al., 1999 | Phonological and Semantic Verbal Fluency | 43 | 19 | 63.40 | 18.00 | 24 | 38.90 | 24.10 |

*Abbreviations: TRS* = treatment resistant schizophrenia; *BACS* = Brief Assessment of Cognition in Schizophrenia; *BRCCB* = Brain Resource Centre Cognitive Battery; *CANTAB* = Cambridge Neuropsychological Testing Automated Battery; *CI* = confidence interval; *FSIQ* = Full scale IQ; *MCCB* = MATRICS Consensus Cognitive Battery; *WAIS-III* = Wechsler Adult Intelligence Scale-Third edition*; WAIS-R* = Wechsler Adult Intelligence Scale- Revised; *WASI* = Wechsler Abbreviated Scale of Intelligence

* Performance scores were inverted prior to analysis to reflect a consistent direction of impairment across cognitive measures.

^1^ The main analysis included all treatment-resistant schizophrenia (TRS) samples across publications, except for the clozapine-resistant samples in Anderson et al., 2015 and Lin et al., 2019. Both clozapine-resistant samples were added to the main analytic sample as part of our sensitivity analysis (see Supplementary Figure S.3).

^2^ The sub-analysis focused selectively on language-related functions that were extracted from across the primary cognitive domains of the main analysis, in addition to Wechsler Vocabulary; the latter task was only included in the sub-analysis and did not feature in the main analysis.

**Table S.5**

*Meta-regression analysis for cognitive domains that showed moderate to substantial heterogeneity of effect sizes in the main analysis and sub-analysis*

| Cognitive domain | Variable | Coefficient | SE | T | P-value | L95%CI | U95%CI |
| --- | --- | --- | --- | --- | --- | --- | --- |
| Executive function | *Diff* Age | .065 | 0.10 | 0.67 | .524 | -0.16 | 0.29 |
|  | *Diff* Duration of illness | .022 | 0.06 | 0.38 | .720 | -0.12 | 0.17 |
|  | *Diff* Age of onset | .025 | 0.22 | 0.11 | .913 | -0.54 | 0.59 |
|  | *Diff*  % Males | - | - | - | - | - | - |
|  | *Diff* Years of education | .226 | 0.25 | 0.91 | .396 | -0.38 | 0.83 |
|  | *Diff* Chlorpromazine equivalents | < -.001 | < .001 | -0.52 | .622 | < -.001 | < .001 |
|  | *Diff* Positive symptom ratings (z score) | .048 | 0.15 | 0.32 | .764 | -0.34 | 0.44 |
|  | *Diff* Negative symptom ratings (z score) | .051 | 0.29 | 0.18 | .866 | -0.66 | 0.76 |
| Verbal memory and learning | *Diff* Age | .033 | 0.14 | 0.24 | .822 | -0.33 | 0.39 |
|  | *Diff* Duration of illness | .083 | 0.21 | 0.40 | .712 | -0.50 | 0.67 |
|  | *Diff* Age of onset | -.152 | 0.17 | -0.88 | .443 | -0.70 | 0.40 |
|  | *Diff*  % Males | -.050 | 0.07 | -0.71 | .530 | -0.27 | 0.17 |
|  | *Diff* Years of education | .340 | 0.17 | 2.00 | .116 | -0.13 | 0.81 |
|  | *Diff* Chlorpromazine equivalents | -.002 | 0.00 | -1.96 | .108 | -0.00 | 0.00 |
|  | *Diff* Positive symptom ratings (z score) | .327 | 0.86 | 0.38 | .728 | -2.40 | 3.05 |
|  | *Diff* Negative symptom ratings (z score) | -.519 | 1.18 | -0.44 | .682 | -3.79 | 2.75 |
| Language function | *Diff* Age | -.064 | 0.09 | -0.71 | .515 | -0.31 | 0.19 |
|  | *Diff* Duration of illness | -.020 | 0.11 | -0.18 | .872 | -0.49 | 0.45 |
|  | *Diff* Age of onset | -.052 | 0.20 | -0.27 | .808 | -0.67 | 0.57 |
|  | *Diff*  % Males | .031 | 0.03 | 1.14 | .317 | -0.04 | 0.11 |
|  | *Diff* Years of education | .503 | 0.23 | 2.16 | .097 | -0.15 | 1.15 |
|  | *Diff* Chlorpromazine equivalents | .001 | 0.00 | 0.67 | .552 | -.0.00 | 0.00 |
|  | *Diff* Positive symptom ratings (z score) | -.253 | 0.16 | -1.61 | .248 | -0.93 | 0.42 |
|  | *Diff* Negative symptom ratings (z score) | -.491 | 0.24 | -2.07 | .130 | -1.24 | 0.26 |

**Figure S.1**

*Forest plots of effect sizes of performance differences in language-related functions between treatment-responsive and treatment-resistant patients (sub-analysis)*

**

**Figure S.2**

*Sensitivity analysis:* *Forest plot of effect sizes of performance differences between treatment-resistant and treatment-responsive schizophrenia patients after excluding clozapine-responsive TRS samples (Anderson et al., 2015; Lin et al., 2019)*

**Figure S.3**

*Sensitivity analysis: Forest plot of effect sizes of performance differences between treatment-resistant and treatment-responsive schizophrenia patients after adding^1^ clozapine-resistant TRS samples (Anderson et al., 2015; Lin et al., 2019)*

**

^1^ To approximate the predominant sampling strategy in the research literature (which is based on samples unselected for clozapine response), and to preserve the independence of samples within meta-analyses, the cognitive scores of the clozapine resistant TRS patients in Anderson et al. (2015) and Lin et al. (2019) were averaged with those of their clozapine responsive counterparts before adding the full samples in the meta-analyses.
